# A Comparative Analysis of Three Non-Invasive Human-Machine Interfaces for the Disabled

**DOI:** 10.3389/fnbot.2014.00024

**Published:** 2014-10-27

**Authors:** Vikram Ravindra, Claudio Castellini

**Affiliations:** ^1^Robotics and Mechatronics Center, German Aerospace Center (DLR), Weßling, Germany

**Keywords:** pressure sensing, machine learning, incremental learning, human–machine interaction, rehabilitation robotics, force control

## Abstract

In the framework of rehabilitation robotics, a major role is played by the human–machine interface (HMI) used to gather the patient’s intent from biological signals, and convert them into control signals for the robotic artifact. Surprisingly, decades of research have not yet declared what the optimal HMI is in this context; in particular, the traditional approach based upon surface electromyography (sEMG) still yields unreliable results due to the inherent variability of the signal. To overcome this problem, the scientific community has recently been advocating the discovery, analysis, and usage of novel HMIs to supersede or augment sEMG; a comparative analysis of such HMIs is therefore a very desirable investigation. In this paper, we compare three such HMIs employed in the detection of finger forces, namely sEMG, ultrasound imaging, and pressure sensing. The comparison is performed along four main lines: the accuracy in the prediction, the stability over time, the wearability, and the cost. A psychophysical experiment involving ten intact subjects engaged in a simple finger-flexion task was set up. Our results show that, at least in this experiment, pressure sensing and sEMG yield comparably good prediction accuracies as opposed to ultrasound imaging; and that pressure sensing enjoys a much better stability than sEMG. Given that pressure sensors are as wearable as sEMG electrodes but way cheaper, we claim that this HMI could represent a valid alternative/augmentation to sEMG to control a multi-fingered hand prosthesis.

## 1 Introduction

Despite decades of research by roboticists, mathematicians, and physiatrists, properly controlling a hand prosthesis by a hand amputee is, still nowadays, an open problem. In the ideal case, proper control means that each single degree of freedom (DOF) of the prosthesis should be independently and proportionally controllable according to the amputee’s intent; for instance, the desire to grab a bottle should immediately result in a stable cylindrical power grasp. So far, this is only enforced on single-DOF grippers such as, e.g., Otto Bock’s Sensorhand Speed^1^ using two surface electromyography (sEMG) electrodes (Merletti et al., 2009, 2011a,b) placed on the *loci* of maximal residual muscle activity found on the stump. Although this form of control is reliable, it cannot go very much past such non-dexterous prostheses, e.g., proportional control of two DOFs at best, with switching between DOFs enforced by sequences of cocontractions.

On the other hand, with the recent advent of multi-fingered hand prostheses as well as active prosthetic wrists and elbows, the need for more dexterous control has become urgent. Commercially available devices (certified as prostheses and employed in the clinics) include, e.g., Touch Bionics’s *i-LIMB Ultra Revolution*, RSL Steeper’s *BeBionic*, and Vincent Systems’s *Vincent Evolution 2*^2^, each one equipped with four to six motors and single-finger DOFs; in some cases, the prosthesis can even independently rotate and flex the thumb. Academic prototypes are going in the same direction, e.g., Prensilia’s *Azzurra* hand, derived from the SmartHand (Cipriani et al., 2011), gifted with five independent motors and tendon-actuated fingers, and the SoftHand (Catalano et al., 2012), exploiting the concept of motor synergies (Santello et al., 1998) to dramatically simplify the control without reducing the functionality. For these mechatronic artifacts, Amsüss et al. (2014) have shown that the old one-DOF control schema does not suffice any longer; more sophisticated forms of sEMG-based control, relying on pattern matching, do not yet work as desired. Matching sEMG patterns via classification is clumsy, unstable, and limiting for the patient, to the point that (Micera et al., 2010; Peerdeman et al., 2011) a large percentage of hand amputees do not routinely use such costly devices.

The community is therefore calling for novel human–machine interfaces (HMIs) to complement, augment, or substitute sEMG, together with the necessity to enforce simultaneous and proportional control using these signals (Fougner et al., 2012; Jiang et al., 2012). To this aim, novel HMIs are currently being explored (Castellini et al., 2014) for control of multi-fingered hand prostheses, but so far little is known about their comparative advantages and disadvantages. In this paper, we propose one such analysis focusing on two such HMIs recently appeared in the scientific literature, namely ultrasound imaging (Sierra González and Castellini, 2013) and pressure sensing (Wininger et al., 2008; Yungher et al., 2011), in comparison to sEMG. We analyzed their performance under four aspects: the accuracy in the prediction of the activation of the DOFs of the prosthesis; the stability of the prediction over time; the wearability; and the projected costs.

A psychophysical experiment was set up in which ten able-bodied subjects would repeatedly flex their fingers to different extents of maximal voluntary contraction, while their sEMG signals, pressure signature of the forearm, and ultrasound images of the forearm were recorded. The experimental results seem then to indicate that pressure sensing represents a viable alternative to sEMG, at least as far as single-finger-force control of a hand prosthesis is concerned.

### 1.1 Related work

The three HMIs chosen for this study have already been individually investigated for hand prosthetic control. Namely, sEMG is a very long-standing choice, its use dating back to the 60s for one-DOF control (Finley and Wirta, 1967). Several comprehensive surveys about its use in modern, multi-DOF hand prostheses have appeared (e.g., Micera et al., 2010; Peerdeman et al., 2011), showing that basically all possible electrode arrangements and machine-learning methods have been tried to enable fine control over self-powered prosthetic hands; nevertheless, it turns out that sEMG has a number of drawbacks, which still make it unsuitable. The main problem lies in its changing nature due to sweat, electrode shift, and fatigue (Merletti et al., 2011a), the latter being especially hard to counter since it entails shifts in the frequency components of the signals as well as in its amplitude. The problem is all the more relevant whenever standard commercial electrodes, such as, e.g., Otto Bock’s MyoBock 13E200^3^ are used, which are the *de facto* standard in clinical applications. These electrodes provide a rectified, low-pass filtered version of the raw sEMG signal, with no original frequency content remaining. Attempts to counter fatigue include, e.g., the usage of switching regimes as the signal changes (Artemiadis and Kyriakopoulos, 2011) or static methods, i.e., filtering (Dimitrova and Dimitrov, 2003). The limited success of these methods mean that the level of abandonment of hand prostheses remains unusually high (Biddiss and Chau, 2007; Peerdeman et al., 2011).

The idea of using ultrasound imaging is, as well, not new. Zheng et al. (2006) originally used it to track the thickness of the extensor muscle in the forearm to drive a one-DOF prosthetic wrist; the approach was then extended in Chen et al. (2010, 2011) and Shi et al. (2010) to multiple DOFs and to a better accuracy. Castellini et al. (2012) and Sierra González and Castellini (2013) demonstrated its feasibility to predict finger forces and positions in a realistic setting, i.e., using no sensors for ground truth and training on maximal and minimal forces only. An attempt at comparing sEMG and ultrasound imaging in a discrete tracking task appears in Jing-Yi et al. (2011), from which it seems that ultrasound imaging obtains better results than sEMG. In Castellini (2014), it is speculated that, given the wealth of information obtained from ultrasound images, this technique might be superior to sEMG.

As far as pressure sensing is concerned, the main idea is that of detecting the intent of a subject by estimating the deformation induced on the forearm/stump surface by the underlying muscle activity – the so-called “pressure signature” of the muscle activation. This path was initially explored in the 2000s by Craelius and Wininger (Craelius, 2002; Phillips and Craelius, 2005; Wininger et al., 2008) who showed that an array of variable resistors could be used to determine the forces required during walking and/or while grasping a dynamometer. Comparison between sEMG and this technique showed that these novel signals enjoy less variability in time and provide more repeatable patterns. This is not surprising, since muscular fatigue should not affect such signals. In Castellini and Ravindra (2014), the effectiveness of such an approach was further demonstrated by showing that a simple bracelet containing 10 force-sensing resistors (FSRs), similar to those used by Craelius and Wininger, can be used to detect finger forces to a degree of accuracy comparable to that found in literature for sEMG.

In the following, we will denote the three above-described HMIs as, in turn, sEMG (surface electromyography), US (ultrasound imaging), and FSR (the acronym of force-sensing resistors, the devices used to gather the pressure signature of the forearm).

## 2 Materials and Methods

A psychophysical experiment involving ten able-bodied subjects was set up, in order to determine the pros and cons of the three HMIs mentioned above. We gathered fingertip forces from the subjects, while they were induced to apply force patterns using a visual stimulus. At the same time, their sEMG signals, FSR signals, and US images of the forearm were recorded.

### 2.1 Experimental setup

#### 2.1.1 Forearm pressure signature

**Figure 1:**
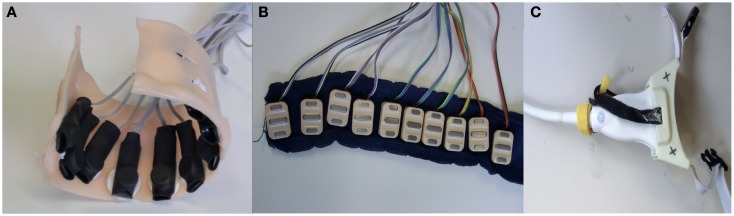
**HMI devices used: (A) customized arrangement of FSR housed in a semi-rigid bracelet; (B) ten sEMG electrodes arranged on a strip of bio-compatible self-adhesive tape; (C) ultrasound transducer fixed to a custom-made cradle**.

The pressure sensing setup comprised a semi-rigid bracelet with ten force-sensing resistors (*FSR400 short* by Interlink Electronics^4^ affixed along the inner surface of the bracelet, as shown in Figure 1A). The sensors have a 5.6 mm-diameter pressure sensitive surface, whose resistance changes in a predictable manner when a force is applied on it. The behavior of the sensor is typically non-linear, with no guarantee of repeatability across sensors, which accounts for their inexpensiveness – a single unit costs in Germany <5 EUR. Each force sensor was placed on an aluminum plate of 3 cm × 1 cm × 1 cm dimension and immobilized by means of a double sided tape. A spherical rubber foot was placed on the sensing surface and the whole arrangement was encased in a heat shrink rubber tubing so as to keep the parts in place. A detailed description of the bracelet as well as of its calibration can be found in Castellini and Ravindra (2014).

#### 2.1.2 Surface electromyography

Ten *Ottobock MyoBock* 13E200 sEMG electrodes were attached to a bio-compatible reusable self-adhesive tape at regular intervals – see Figure 1B. These electrodes are the standard, off-the-shelf sEMG device used in clinical prosthetic sockets, and are commercially available; they provide an amplified, bandpass filtered, rectified sEMG signal, of excellent quality for prosthetic control. The electrode band was placed around the forearm, about 5 cm below the elbow; no muscle targeting was enforced in order to keep the electrode positioning as simple as possible. This choice has already been proved effective for hand prosthetic control (Castellini et al., 2009; Castellini and van der Smagt, 2013). Each such electrode can be bought at a price between 150 and 300 EUR in Europe.

#### 2.1.3 Ultrasound imaging

Ultrasound images of the forearm were gathered using a General Electric *Logiq-e* portable ultrasound machine^5^ equipped with a 12L-RS linear transducer. Movement of the probe with respect to the subject’s skin was avoided using a custom-built plastic cradle obtained via rapid prototyping – see Figure 1C. The machine was configured to an ultrasound frequency of 12 Hz, edge enhancement on, focus point at a depth of 1.3 cm and minimum depth of field, resulting in a frame rate of 38 Hz. For a deeper description of this setup, please refer to Sierra González and Castellini (2013).

#### 2.1.4 Finger-force sensor

The Finger-Force Linear Sensor (FFLS, see Kõiva et al., 2012) was used to gather the applied finger forces. The FFLS is a customized arrangement of six strain gage sensors, ergonomically designed to fit the positions of fingers while the palm is stretched. It employs four *KD60-100N* industrial strain gage sensors by ME-Meßsysteme GmbH^6^ to measure finger flexion and extension forces of the index, ring, middle, and little fingers, and one *RFS*^®^ *150XY-100-8-3* dual axis strain gage sensor by Honigmann GmbH^7^ to measure the flexion/extension and abduction/adduction forces of the thumb. The sensors have <0.1% linearity error and 0.1% drift over 30 min. The FFLS guarantees therefore a repeatable linear behavior in a high range of force (100 N in all directions) and reaches an overall accuracy of +0.35% over nominal measurement range.

### 2.2 Experiment description

An experiment was designed to compare the accuracy and stability of the finger-force prediction obtained by a state-of-the-art machine-learning method using the three kinds of signals provided by each HMI. In an initial round of data collection, we measured the maximal forces that each subject could apply at each of the 6 DOFs considered (namely flexion of the little, ring, middle, and index finger plus thumb adduction and flexion). The choice of these six DOFs of the human hand is motivated by the fact that one of the most dexterous self-powered hand prostheses in the world at the time of the experiment, namely the *i-LIMB Ultra Revolution*, has exactly those active six DOF available; and it is reflected in the configuration of the FFLS, which can measure the forces applied for each of these DOFs.

**Figure 2:**
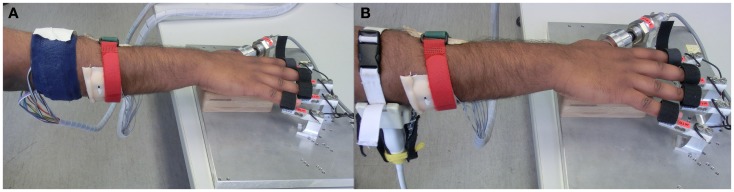
**Experimental setup comprising HMIs on the forearm and the finger tips on the FFLS**. **(A)** FSR bracelet and sEMG band; **(B)** FSR bracelet and US transducer applied to the forearm.

In the ideal case, data should have been collected with all three devices strapped to the forearm at the same time. However, such an arrangement would have seriously hampered the comfort of the subjects. We rather did a pairwise comparison of the FSR with sEMG and US. This pairwise comparison was done by dividing the data collection into two phases. In the first phase, the sEMG band and the FSR bracelet were affixed to the right forearm of the subject (Figure 2A); whereas in the second phase, the sEMG band was replaced by the ultrasound probe (Figure 2B). The ground truth was collected by strapping the fingertips on the sensors of the FFLS.

In both phases, the same visual stimuli were administered to the subjects: a 3D hand model of a human hand, flexing the fingers, plus a colored bar denoting the required level of force. A further set of colored bars denoting the force actually applied by the subjects helped ensure that the subjects would exert the required amounts of force to their best. The two phases were identical in terms of the actions that were required by the subject. Each phase comprised ten *repetitions*, each repetition consisting of a single-finger flexion for each DOF. During repetitions 1–5, the subjects were required to flex their fingers at 80% maximum force level; the subject was then given a break of 5 min, followed by repetitions 6–10, at 15% of maximum force. This split provided an indication of how the prediction accuracy would behave in a high-force as well as in a low-force scenario.

Ten able-bodied subjects volunteered for the experiment. The entire experimental procedure was conveyed to them in oral and written form, following which their consent was taken in writing. The experiment was approved by the Ethical Committee of DLR. The subjects were seated comfortably on an adjustable office chair for the entire duration. They were given the option to pause or stop the experiment at any point of time.

### 2.3 Data processing and force prediction

Data obtained from the sEMG, FSR, and FFLS were collected at 50 Hz using a standard analogic-to-digital conversion card. The sEMG signals were filtered by a 1st order low-pass butterworth filter with cutoff frequency at 1 Hz. A gradual drift was observed in the FSR signals, which was remedied by applying a 3rd order high-pass Butterworth filter with cutoff frequency at 0.5 Hz. The filtered signals obtained from the sEMG electrodes and FSRs were directly used, without any further feature extraction phase. The US images were grabbed from the VGA output (at 38 Hz) of the US machine using a standard PCI-bus frame grabber; from each image, 543 local visual features were extracted, denoting the changes in the local levels of gray of each image. For a thorough description of the feature extraction procedure, please refer to Sierra González and Castellini (2013).

Following the approach tested in the same paper, we applied Ridge Regression to the visual features in order to predict the finger forces. Ridge Regression (Hoerl and Kennard, 1970) is a regularized variant of least-squares regression, building a linear model of the forces of the form *f* (*x*) = *w^T^x*, where *x* denotes the visual features extracted from each image, and *w* is a weighting vector, which can be calculated in closed form from a set of (sample, target) pairs (the *training set*) previously collected. In particular, if *X* denotes the training set (as an *n* × 543 matrix, where *n* is the number of samples in the training set), and *y* is a vector collecting the *n* target (force) values for each collected sample, then *w* = (*X^T^X* + λ*I*)^−1^*X^T^y*. (The regularization coefficient λ was set at the standard value of 1.)

For sEMG and FSR data, a linear approach is not sufficient (Castellini and Ravindra, 2014; Gijsberts et al., 2014), therefore we employed a non-linear extension to Ridge Regression called Random Fourier Features (Rahimi and Recht, 2008). The models obtained by this approach have the form *f* (*x*) = *w^T^ϕ*(*x*), where *ϕ* is a non-linear mapping from the input space (the sEMG or FSR values) to a higher- (but finite-) dimensional *feature* space, where linear regression is more likely to succeed. The mapping *ϕ* is actually a finite approximation of the well-known radial-basis-function kernel; but, as opposed to what happens, e.g., with Support Vector Machines (Boser et al., 1992), where the kernel is known but the mapping is not, leading to an infinite-dimensional feature space, in this case *ϕ* can be explicitly (and computationally lightly) evaluated. The finiteness of the induced kernel keeps the approach bounded in space and permits the mapping to simply be “plugged in” into the Ridge Regression formula: *w* = (*ϕ*(*X*)*^T^ϕ*(*X*) + λ*I*)^−1^*ϕ*(*X*)*^T^y*. More details about the theory of this method when applied to sEMG can be found in Gijsberts et al. (2014).

The prediction accuracy of each machine-learning method (Ridge Regression and Random Fourier Features in turn) was evaluated by following the *realistic scenario* first described in Sierra González and Castellini (2013): to build a training set, only data collected during the application of maximal and minimal forces were considered; fivefold cross-validation was done for each subject, by training on four out of five repetitions and testing on the remaining repetition. In the case of Random Fourier Features, the hyperparameter *σ* was found by grid-search while the number of features to be evaluated, *D*, was set at the computationally feasible value of 300. Training sets were built, in turn, using either the FFLS force data (the “ideal” ground truth) and the stimulus values – a harder but more realistic choice, since amputees cannot provide any consistent ground truth using sensors. The prediction accuracy of each machine-learning method applied to either regression problem (with forces or stimulus as ground truth) was evaluated by calculating the Root Mean-Squared-Error between the predicted force values and the ground truth obtained from the FFLS sensor, normalized over the target signal range (nRMSE). Notice that this problem represents a hard challenge for any machine-learning method whatsoever: in the most difficult setting, the system is trained on stimulus values *but still tested on real force data*.

Lastly, two prediction scenarios were considered, one to ascertain the accuracy obtained by each HMI in general, and another one to test the stability over time of each HMI. For the first scenario, the above cross-validation procedure was used; for the second, we trained on data collected during the first repetition, and subsequently tested on the second, third, fourth, and fifth repetition separately. As the repetitions were always collected in the same chronological order, this would give us an indication of how well the prediction could carry on over time.

## 3 Results

### 3.1 Prediction accuracy

**Figure 3:**
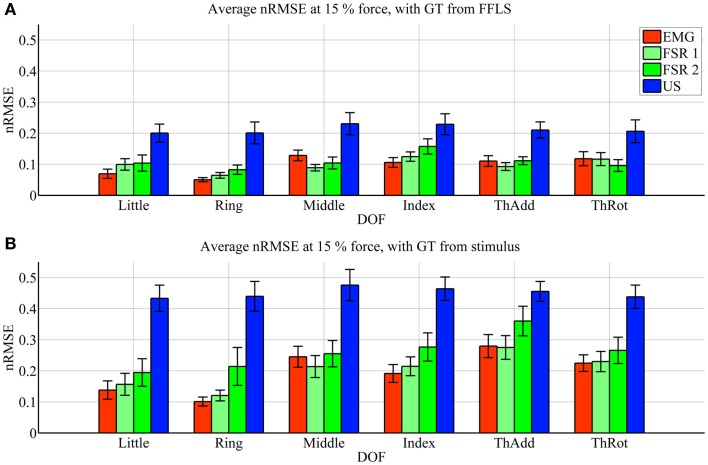
**Prediction accuracy (nRMSE) obtained by each HMI for each degree of freedom considered, during the high-forces experiment (stimulus at 80% of the maximum voluntary contractions)**. **(A)** Error obtained when the FFLS data are used as ground truth; **(B)** error obtained when the stimulus is used as ground truth. The legend denotes, in turn, sEMG (EMG), the two FSR sessions (FSR1, FSR2) and ultrasound imaging (US). Bars and stems denote average nRMSE values across ten subjects, plus/minus one standard error of the mean.

**Figure 4:**
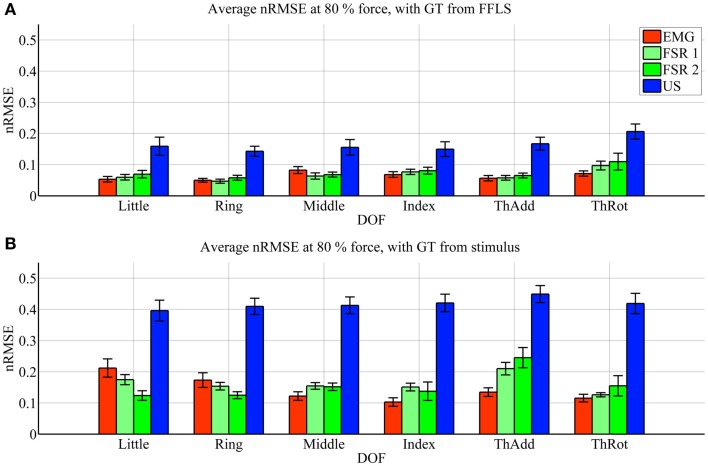
**Prediction accuracy (nRMSE) obtained by each HMI for each degree of freedom considered, during the low-forces experiment (stimulus at 15% of the maximum voluntary contractions)**. **(A)** Error obtained when the FFLS data are used as ground truth; **(B)** error obtained when the stimulus is used as ground truth. The legend denotes, in turn, sEMG (EMG), the two FSR sessions (FSR1, FSR2) and ultrasound imaging (US). Bars and stems denote average nRMSE values across ten subjects, plus/minus one standard error of the mean.

Figures 3 and 4 show the prediction accuracy obtained by each HMI on each degree of freedom considered (little, ring, middle, and index finger flexion, thumb adduction, and thumb rotation), in the high- and low-forces experiment, in turn, both when the FFLS (force) data are used as ground truth (Figures 3A and 4A) and when the stimulus values are used to this purpose (Figures 3B and 4B).

In the graphs, FSR1 and FSR2 denote the two identical experiments performed with FSR, the first time together with sEMG and the second with US. Almost no statistically significant difference in performance is observed (Student’s two-tailed *t*-test *p*-value always larger than 0.05, except for one single case). This confirms that the error values for the FSR obtained in the two experiments are similar, in turn, confirming that the subjects were induced to do approximately the same things during the two experiments. The maximum force exerted averaged over ten subjects and the six degrees of freedom is 22.6 ± 2.1 N.

As far as the prediction accuracy is concerned, in the high-forces case, when the actual forces (FFLS data) are used as ground truth to train the system (Figure 3A), the error attained by sEMG and FSR is almost uniformly below 10%, with very few statistically significant differences. Ultrasound imaging obtains a rather higher error, in the range 15–20%, always statistically significant. In case the stimulus values are used as ground truth for training (Figure 3B), the situation gets uniformly worse as expected, but the same pattern can be observed: sEMG and FSR obtain errors in the range 10–20% (around 20% in the case of thumb adduction), which corresponds to a maximum error of approximately 4.3 N, while ultrasound imaging performs overall much worse, with errors hovering around 40%, which is approximately 7.2 N. Again, its performance is always statistically significantly different in comparison to the worse among the other approaches.

In the low-forces experiment (Figure 4), the same considerations hold, given the fact that the error becomes uniformly higher. This is to be expected, since the range of forces in this case is significantly smaller than in the high-forces case. It should be noted that though the nRMSE values obtained with sEMG and FSR is high for some DOFs (20–35% for middle, index, and thumb adduction/rotation), the minuteness of the 15% force level means that the error in terms of physical force is approximately 1.2 N.

### 3.2 Stability

**Figure 5:**
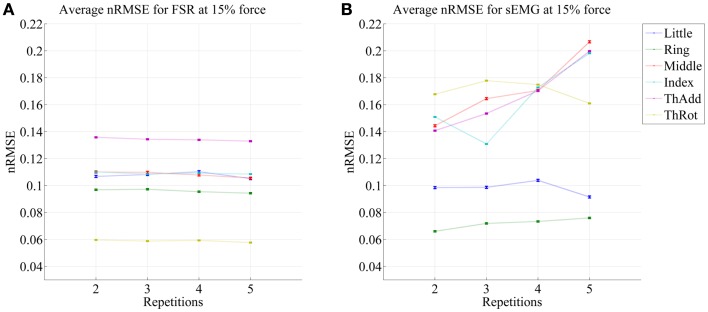
**Prediction accuracy (nRMSE) obtained by FSR (A) and sEMG (B) for each degree of freedom considered, during the high-forces experiment (stimulus at 80% of the maximum voluntary contractions); the system was trained on the first repetition; the graph shows the error obtained while testing on repetitions #2, #3, #4, and #5**. The FFLS data are used as ground truth.

**Figure 6:**
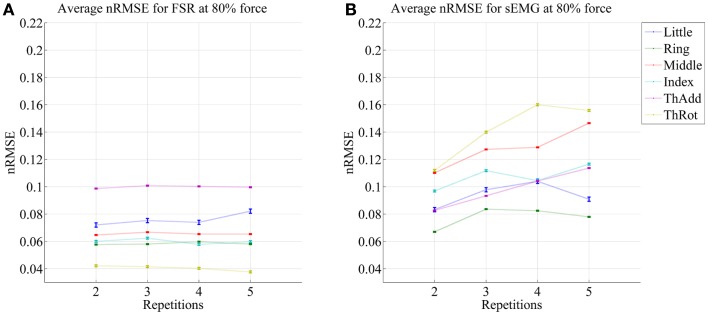
**Prediction accuracy (nRMSE) obtained by FSR (A) and sEMG (B) for each degree of freedom considered, during the low-forces experiment (stimulus at 15% of the maximum voluntary contractions); the system was trained on the first repetition; the graph shows the error obtained while testing on repetitions #2, #3, #4, and #5**. FFLS data are used as ground truth.

Figures 5 and 6 show the prediction errors obtained by FSR and sEMG on each degree of freedom considered, in the high- and low-forces experiment, in turn, split across repetitions #2, #3, #4, and #5. Training was done on the first repetition only. Testing was done on the FFLS ground truth only, to obtain a clearer picture.

From the figures, a clear trend up is evident in the case of sEMG, all the more in the case of the low-forces experiment. In fact, the slope of a linear fit (averaged over the six degrees of freedom) turns out to be 0.0002 ± 0.0014 and −0.0008 ± 0.0004 for FSR, and 0.0079 ± 0.0005 and 0.0092 ± 0.0105 for sEMG. This confirms that FSR enjoys a better stability in time with respect to sEMG.

## 4 Discussion

The experiment described in this paper is a comparative analysis among HMIs for the disabled. Particular care was taken to ensure that the three HMIs under examination were tested in the very same conditions. Since it was infeasible to have the subjects wear the sEMG electrodes, the pressure sensing bracelet and the ultrasound imaging probe all at the same time, we rather tested the HMIs in two pairs, checking later on that the common one (namely, the tactile bracelet) would obtain comparable results in the two experiments.

The experimental results show that, overall, all three HMIs can be in principle used in a realistic setting – that is, using an incremental, real-time machine-learning method, in principle trained without the usage of sensors for ground truth, and using a very short training procedure (on minimal and maximal forces only). All three HMIs can potentially enforce simultaneous and proportional control over fingers of a multi-fingered prosthesis. A more detailed discussion of the results follows.

### 4.1 Prediction accuracy

Ultrasound imaging provided a surprisingly unsatisfactory performance. According to Figures 3 and 4, the nRMSE achieved by ultrasound imaging in the most difficult settings (that is, when training on the stimulus as ground truth) hovers around 40% both in the high- and low-forces experiment. This is a surprisingly high value if compared to previous work (Sierra González and Castellini, 2013). It must be noted, however, that in that work, the cross-validation was done by random shuffling of all samples gathered in one series of finger-force repetitions; this is of great aid to any machine-learning method, since it represents a uniform sampling of the whole probability distribution of the data. As opposed to that, in this experiment the cross-validation is performed *repetition-wise*, that is, by training on four out of five repetitions, and then testing on the remaining repetition. It can therefore be the case that the ultrasound visual features extracted from each repetition slightly differ from one another (due to probe shift or different levels of muscle activation and shifting from one repetition to the next one). In an initial round of experiments on the same data set performed using random shuffling in cross-validation, error values similar to those obtained in Sierra González and Castellini (2013) have appeared, confirming the above claim.

Surface electromyography and pressure sensing, on the other hand, perform better. In the same hard setting, both sEMG and pressure sensing achieve errors in the range 10–20% at high-force level, in line with existing literature (for example, Castellini and Ravindra, 2014; Gijsberts et al., 2014). Though the accuracy, in terms of nRMSE, deteriorates at low-force level, it maps onto a force error of just 1.2 N. A direct comparison between these two HMIs, performed using Student’s *t*-test, reveals very few significant differences in performance.

### 4.2 Stability over time

It is well-known (Merletti et al., 2011a) that the sEMG signal suffers from non-trivial substantial changes over time during stress conditions; this is due to electrode displacement, muscular fatigue, and the formation of sweat at the interface between the electrodes and the skin. An HMI based upon pressure sensing, on the other hand, should not suffer from this problem, as the forearm deformations induced by the underlying muscular activity should still reflect the actual activations. Our stability analysis confirms this claim, showing that (consider now Figures 5 and 6) the prediction error achieved by sEMG rapidly degrades over time in both settings, going from 7–11 to 8–14% in the high-forces case and from 7–15 to 8–20% in the low-forces one. This is an ominous problem, already highlighted and studied in previous work (Gijsberts et al., 2014); the solution that we proposed and positively tested in that paper was that of enforcing an *incremental* learning system, so that whenever required, prediction errors could be amended by inserting new knowledge in the existing models. (This is as well the main motivation for using, in this very work, Ridge Regression with and without Random Fourier Features.)

As opposed to this degradation suffered by sEMG, the prediction obtained by pressure sensing remains stable at 4–10 and 6–14%. This result is in line with former literature (Yungher et al., 2011) and provides an indication that pressure sensing might be a viable companion to (or even a substitute of) sEMG, when stability over time is an issue. Notice that high-resolution tactile sensing, an approach very closely related to ours, is already being investigated as a means of intent detection for the disabled (Radmand et al., 2014).

### 4.3 Physiological considerations

Due to the mainly applicative focus of this work, the analysis presented in this paper neglects almost all physiological aspects of machine-learning-based intent detection/prosthetic control; in particular, no physical data have been recorded for the subjects (e.g., the forearm girdle and length, the muscle fitness, etc.), and the analysis has been performed by finding the optimal machine-learning hyperparamters either by referring to previous literature or by extensive (grid-) search (see Subsection 3 again). In other words, each model found (and the associated prediction accuracy) is the optimal one for the related subject and dataset, *irrespective of her/his physical condition and anatomical features*. This is nowadays a standard procedure in rehabilitation robotics, since the aim is that of providing the best precision and effectiveness to any human subject who needs to use the system. With this aim in mind, we have adopted the “realistic setting” detailed in Sierra González and Castellini (2013), in which the machines are trained on minimal and maximal stimulus values only, since amputees cannot produce force ground truth nor precisely follow graded stimuli. It is, however, worth to stress once again that the performance testing has been done *on the full range of forces*, including the intermediate ones. This means that the prediction performance is the one, which would be obtained on average, on the full range of required forces.

Notice, moreover, that disabled subjects usually have abnormal physical/psychophysical parameters with respect to the healthy population; for example, Parkinson patients present a higher amount of non-voluntary muscle activation than control subjects (Torres et al., 2011). In the case of transradial amputees, who are the focus population of this research, the stump can hardly be related to the intact human forearm, especially when the amputation is proximal (Watson et al., 2010), a neuroma is formed and, after the operation, muscles attach to the stump ending, the neuroma itself or the bone stump. This abnormal reorganization almost prevents the detection of standard muscle activity patterns in such patients; nevertheless, in most studies in which amputees have tested systems such as the one that we have presented here, it was clear that enough information was still present to reach remarkable levels of control, sometimes to the level of single fingers (Tenore et al., 2009). To ensure a reliable prediction, machine-learning methods require repeatable and distinct patterns corresponding to the desired activations, potentially irrespective of their physiological meaning.

### 4.4 Wearability and cost

A few qualitative considerations on the wearability and costs of the three examined HMIs follow. Ultrasound imaging is, as yet, not wearable. To the best of our knowledge, neither B-mode ultrasound interface has so far been realized that can be embedded into a prosthetic socket nor any array of ultrasound transducers has been built, that can be embedded in a flexible structure (e.g., a Lycra sock or glove) to be worn on the skin. The only attempt we are aware of so far is represented by Guo et al. (2013), where one single transducer was affixed to a subject’s forearm, this way realizing a very preliminary form of wearable A-mode ultrasonography. It must be noted, however, that a really fully wearable US system calls for a quite radical advancement in the technology, since the electronics required to form the beam of ultrasound waves is much more complex than that required for gathering a set of voltages. In addition to this, the long-term effects of ultrasound waves on the human tissues are still unknown, which would require a clinical trial (Castellini, 2014). As opposed to this, the wearability of the sEMG and pressure sensing devices has already been shown in a number of works: sEMG is in use in the clinics, the electrodes being embedded in the plastic cast, which support the hand prosthesis; the pressure sensing bracelet that we used here weighs about 65 g and requires no preparation at all (Castellini and Ravindra, 2014).

As far the costs are concerned: cheap as it has become in recent times with respect to the past years (a hand-held ultrasound machine can, at the time of writing, be purchased for about 8.000 USD), this kind of hardware remains prohibitively expensive to be shipped along with a hand prosthesis, constituting a relevant fraction of the price of the hand itself (hovering around 25.000 EUR). Each sEMG electrode of the kind that we used here is in the range of 150–300 EUR a piece, which makes our array significantly cheaper than an ultrasound device; it must be noted that, probably, even cheaper arrays of sEMG electrodes can be purchased, but in that case, a further signal processing phase (rectification, filtering) must take place before the signal is available to the machine-learning method, raising the cost of the whole approach. The pressure sensing bracelet, in turn, cost an estimated 50 EUR as it employs force-sensing resistors, which can be bought for <5 EUR apiece (Castellini and Ravindra, 2014).

### 4.5 Perspectives

To summarize, Table 1 shows a qualitative comparison of the three HMIs (surface electromyography, pressure sensing, and ultrasound imaging) considered from the four points of view that we described above: prediction accuracy, stability over time, wearability, and cost.

**Table 1:** **Qualitative summary of the characteristics of each HMI, denoted as bad/poor/average/good/excellent**.

	Error		Stability		Wearability	Cost
	High forces	Low forces	High forces	Low forces	
sEMG	Good	Good	Poor	Poor	Excellent	Poor
Pressure	Good	Good	Good	Good	Excellent	Excellent
Ultrasound	Bad	Bad	–	–	Bad	Bad

From this comparison, pressure sensing is favored, as it provides the best accuracy (comparable to that of surface electromyography), but it also keeps a remarkably stable prediction over time (as opposed to sEMG). It is also cheaper than sEMG, while maintaining full wearability. This does not mean, of course, that pressure sensing is a definitive replacement for sEMG and/or ultrasound. We speculate that this novel HMI will suffer from a number of disturbances once it is used in a real-life application; in particular, its signal will change as the patient swings the forearm (due to the acceleration of the forearm against the sensors harness) and, which is more worrisome, as (s)he lies the forearm on a surface. These problems will need to be countered; one possibility is to fuse this signal with that obtained from sEMG, which will be much less influenced by such artifacts.

The weak performance of ultrasound imaging in this experiment, together with its low scores as far as cost and wearability are concerned, pose serious doubts on its use as a competitor HMI. Nevertheless, as we had previously claimed (Sierra González and Castellini, 2013), ultrasound images provide an extremely rich set of information about the status of the forearm; in order to fully exploit it, it could be useful to employ more advanced image processing techniques. If such a technique can be found, ultrasound imaging could still be employed as a virtual-reality HMI in a clinic, in order to rehabilitate, e.g., amputees and stroke patients.

## 5 Conclusion

Inspired by the recent trend in the rehabilitation robotics community, in this paper we have compared three HMIs for control of multi-fingered prosthetic hands. Surface electromyography, pressure sensing, and ultrasoundimaging were tested on ten intact subjects engaged in a simple, repetitive application of single-finger-force patterns; a state-of-the-art machine-learning method was then used to predict the forces using the signals gathered from the HMIs. Our experimental results indicate that pressure sensing represents a cheaper alternative to sEMG, enforcing prediction accuracy comparable to sEMG, better stability in time, and the same wearability as this more traditional HMI.

## Conflict of Interest Statement

The authors declare that the research was conducted in the absence of any commercial or financial relationships that could be construed as a potential conflict of interest.

## Footnotes

^1^http://www.ottobock.com

^2^http://www.touchbionics.com, http://www.bebionic.com and http://www.vincentsystems.de

^3^http://www.ottobock.com

^4^http://www.interlinkelectronics.com

^5^http://www.gehealthcare.com

^6^http://www.me-systeme.de

^7^http://www.honigmann.de
